# Sleep Quality and Spiritual Well-Being in Hemodialysis Patients

**DOI:** 10.5812/ircmj.17155

**Published:** 2014-07-05

**Authors:** Ahmad Ali Eslami, Leili Rabiei, Freidoon Khayri, Mohammad Reza Rashidi Nooshabadi, Reza Masoudi

**Affiliations:** 1 Department of Health Education and Promotion, School of Health, Isfahan University of Medical Sciences, Isfahan, IR Iran; 2 School of Health, Isfahan University of Medical Sciences, Isfahan, IR Iran; 3 Nursing and Midwifery School, Iran University of Medical Sciences, Tehran, IR Iran; 4 School of Pharmacy, Ahvaz Jundishapur University of Medical Sciences, Ahvaz, IR Iran; 5 Nursing and Midwifery School, Shahrekord University of Medical Sciences, Shahrekord, IR Iran

**Keywords:** Hemodialysis, Sleep, Spiritual

## Abstract

**Background:**

Sleep disorders are considered as one of the most important problems in hemodialysis patients, making their everyday life a serious hazard. Sleep quality of hemodialysis patients and consequences of sleep disorders on other aspects of health such as spiritual well-being are important issues.

**Objectives:**

This study examined the relationship between spiritual well-being and quality of sleep in hemodialysis patients in Isfahan, Iran.

**Patients and Methods:**

This study was a correlation research, carried out on 190 hemodialysis patients. Data collection Questionnaires included demographic forms, Pittsburgh sleep quality index (PSQI), and Ellison and Paloutzian spiritual well-being scale. Data were analyzed using descriptive and inferential statistics (Pearson correlation and linear regression analysis) at P < 0.05 significance level, by SPSS software version 18.

**Results:**

Of 190 study participants, 163 (85.78%) with scores more than five index had sleep disturbances and 27 (14.12%) had no sleep disturbance; 3 (1.52%) had mild, 163 (85.78%) moderate, and 24 (12.30%) good spiritual health conditions. Pearson correlation test showed significant relationship between the sleep quality items of Pittsburg and spiritual well-being (P < 0.04, r = 0.149). Through the regression analyses of spiritual health, family, education, financial status, marital status, occupation, and use of sleep medication, the predictive power of these variables was found 0.417% and prediction of spiritual well-being was more than others (ß = 0.209).

**Conclusions:**

Considering bed as one of the most vital physical, mental, and emotional needs, it is very important in mental and spiritual well-being of hemodialysis patients as an influencing factor in mental relaxation and reducing disease tensions. Paying attention to sleep quality and spiritual well-being components of hemodialysis patients in formulating and promoting healthcare programs is recommended.

## 1. Background

Proper function of the urinary system is necessary for survival. When kidneys are unable to excrete metabolic waste and perform self-regulatory functions, it is called renal failure, a major health problem worldwide (1). End-stage renal disease is a clinical condition defined as irreversible loss of endogenous renal function, causing permanent renal replacement therapy (dialysis or transplantation) to prevent life-threatening uremia (2). At least 6% of American adults have chronic renal glomerular filtration rate of less than 60% (chronic kidney disease stages 1 and 2), and are in imminent danger of further progressive decline, and 4.5% are in stages 3 and 4 of chronic kidney disease (3). Chronic kidney disease has risen by 8% over the past five years. The disease prevalence is mostly among patients with end-stage renal diseases and is equal to 400 thousand or more in the USA (2). The incidence of end-stage renal disease in the USA and Iran was reported 330 (1) and 253 per one million people annually, respectively (4).

The disease can affect all ages, but it has a peak incidence between the ages of 62-30 years (2). According to the Statistics Center of Iran, in 2008, renal disease and kidney transplant statistics showed that approximately 29000 patients with chronic renal failure were treated, of which 14000 (48.5%) were on hemodialysis treatments (5, 6). Iranian Society of Nephrology's survey in 2009 showed that the number of hemodialysis patients increased again, as to the date, 16600 hemodialysis patients were under dialysis in 355 dialysis units. This number rose to 40000 in 2012 (4). Taking the consequences of this disease for patients is associated with challenges, making their everyday life a serious hazard. One of the common problems in these patients is sleep disorders. According to the previous studies, over 80% of dialysis patients have experienced these disorders (1, 4), including difficulties in falling asleep, remaining asleep, sleeping too much during the day, and restless legs syndrome (7).

Straub et al. conducted one of the first studies on dialysis patients considering prevalence of sleep problems. They found that 63% of dialysis patients had sleep problems such as lack of sleep, fragmented sleep, and long wake time before sleep in bed (8).

There are several concepts in dealing with the disease problems and stress. One of them is spiritual health, comprised of two dimensions of being and religion. Religion gives a kind of satisfaction through contact with a higher power. In this regard, physical health has been defined as the quest for finding meaning and purpose in life (9). Today, some organizations are responsible for evaluation of health care centers and their accreditation for granting, assessing that whether they meet the spiritual needs of patients. American Psychiatric Association recommends that doctors should ask patients to be religious and have spiritual orientation. These recommendations show that patient care is far beyond the diseases treatment (10).

Most people have spiritual lives. Most of the studies conducted on general population and patients have shown that more than 90% of them believed in spiritual health. Moreover, studies have shown that for up to 94% of patients, physical and spiritual health have been equally important. Most patients want to meet their religious and spiritual needs. In a study carried out on family physicians, 96% of respondents believed that spiritual health was an important health factor. Despite these findings, the patient's spiritual needs are often being neglected (11). Some studies indicated that in people without spiritual health, other aspects of biological, psychological and social functioning or reaching the highest potency could not be possible or work properly. Therefore, they cannot achieve the highest quality of life. Throughout the history, religion, spirituality and health have been combined (12).

Currently, most health models, including spiritual health and the concept of spirituality, are associated with all ages and health domains, which has been an interest for researchers around the world (9, 13). Studies imply on the relationship between spirituality and physical and mental health, as well as promoting adaptation. The results of Litwinczuk et al. study on patients with AIDS showed that meaning of life was based on spirituality; religious people have better life qualities (14). In addition, Stuifbergen et al. study on patients with multiple sclerosis showed that spiritual beliefs and faith were helpful factors in coping with the disease (15). Furthermore, O'Conner et al. in their study realized positive effects of spiritual health on quality of life in patients with leukemia (16). Therefore, spirituality and spiritual health have received little attention, while they seem to be effective on various aspects of patients' lives, as it changed the sleep quality of hemodialysis patients in this study.

## 2. Objectives

Considering the sleep quality of hemodialysis patients as well as consequences of sleep disorders on other aspects of health, researcher designed this study to determine the relationship between spiritual well-being and quality of sleep in hemodialysis patients.

## 3. Patients and Methods

This study was a correlation research on 215 hemodialysis patients referred to Isfahan governmental and referral dialysis centers in 2012. Twenty-five patients were excluded because of incomplete questionnaires and the final 190 were entered to the study through convenience sampling method, according to the following statistical formula:

$$n=\frac{{\left(z_{1}+z_{2}\right)}^{2}(1-r^{2})}{r^{2}}\times 2$$

(z_1_: 95٪ = 1.96; z_2_: 80٪ = 0.84; r = -0.2)

Inclusion criteria were hemodialysis treatment for more than three months, receiving hemodialysis for 3-4 hours per session and more than two times a week, age over 19 years old, independent self-care activities, ability to understand the questions, read and write, and consent to participate in the study. Exclusion criteria included mental and cognitive dysfunction and incomplete questionnaire. Protocol of the project with code number 211196 was approved by the Isfahan University of Medical Sciences Research Ethics Committee in October 2012.

### 3.1. Ethical Consideration

The participants were aware of the purpose and importance of the research and informed written consents were obtained. In addition, the patients were assured that participation was voluntarily and they may quit at any time.

Data were collected first by demographic characteristic questionnaire, Pittsburgh sleep quality index (PSQI), and Ellison and Paloutzian 20-item spiritual well-being questionnaire. Pittsburgh questionnaire is composed of 19 self-report questions plus five questions to be reported by the patients’ caregivers (only applications of the mentioned questions were calculated in scoring). Of the 19 questions, 15 are multiple choices, discussing frequency of sleep problems and subjective sleep quality and four items discuss the in-bed time, waking time, dream incubation period, and sleep duration. Five multiple-choice questions are answered by the patient's partner. This questionnaire has seven domains which are discussed respectively. Each component is scored from 0 (no difficulty) to 3 (severe problem). Points for each question are aggregated to obtain an overall score; the total range is from 0 to 21. Total score of 5 or greater is indicative of significant sleep disturbance. In this questionnaire, question 9 is related to quality of sleep, questions 2 and 5a to long incubation period of sleep, question 4 to sleep duration, questions 5b-5j to sleep disorders, question 6 to sedatives usage, and questions 7 and 8 to daily dysfunction.

Validity and reliability of Pittsburgh sleep quality index was assessed by Osorio et al. Backhaus confirmed the reliability as 85% and validity as 83% (17, 18). Reliability of this index was also confirmed in Iran by Soleimani, with α = 0.87 (19). The second tool was spiritual well-being scale of Ellison and Paloutzian, the 1982 questionnaire, with 10 questions assessing the religious health and 10 evaluating the person's health. Validity and reliability of spiritual well-being index was confirmed by Allahbakhshian in a study entitled "spiritual health and quality of life in multiple sclerosis patients", with α = 0.82 (20). Reliabilities of both questionnaires in this study were confirmed using the split-half test, with α = 0.89 for Pittsburgh sleep quality index and α = 0.81 for spiritual well-being index.

The spiritual well-being score was derived from the sum of these two subgroups, which was considered to range between 120-20. The questions answers were classified as completely disagree to strongly agree with Likert scale of 6 items. In questions 3, 4, 3, 7, 8, 10, 11, 14, 15, 17, 19, 20, completely disagree was scored 1 and in questions 1, 2, 5, 6, 9, 12, 13, 16, 18, completely disagree was scored 6. Finally, spiritual health levels of individuals were divided to three categories: low (20-40), Medium (41-99), and high (100-120).

### 3.2. Statistical Analysis

Data were analyzed using descriptive and inferential statistics (Pearson correlation and linear regression analysis) at P < 0.05 significance level, using SPSS software version 18.

## 4. Results

Socio-demographic information of the participants is presented in Table 1. Kolmogorov–Smirnov test showed normal distribution of data. In fact, 81.1% of the patients in hemodialysis unit had one of the sleep disorders. From 190 participants, Pittsburgh sleep quality score of 163 (85.78%) patients had more than five points, showing sleep disturbances, and 27 (14.12%) patients with scores ≤ 5, had no sleep disturbance. Subscales of Pittsburgh sleep quality indicated the following results: mean ± SD of subjective sleep quality: 1.96 ± 0.99, quality during the incubation period of sleep: 3.25 ± 1.43, sleep duration: 1.29 ± 1.17, sleep efficiency: 2.13 ± 1.19, sleeping medications usage: 1.88 ± 1.55, daily abnormal performance: 1.79 ± 0.95, overall sleep quality: 12.92 ± 2.25.

Furthermore, 3 (1.52%) of the patients had mild (10-19), 163 (85.78%) moderate (20-29), and 24 (12.30%) high spiritual well-being. The majority of patients had a moderate spiritual health.

Pearson correlation test showed significant relationship between sleep quality items of Pittsburg (subjective sleep quality, sleep during the incubation period, sleep duration, sleep quality, sleep disturbances, sleeping medications usage, abnormal daily activities) and spiritual well-being (P < 0.04, r = 0.149) (Table 2). In the regression analysis of spiritual health, family, education, financial status, marital status, occupation, and sleep medications usage, the predictive power was obtained 0.417%. Among these variables, predictive ability of education, spiritual health, financial status, marital status, occupation, and sleep medications usage was significant and their prediction of spiritual well-being was more than others (ß = 0.209) (Table 3).

**Table 1.** Demographic Characteristics of Hemodialysis Patients

Variable	No. (%)
**Sex**	
Male	115 (60.4)
Female	75 (39.6)
**Education**	
Illiterate	65 (33.9)
Less than diploma	84 (44.5)
Diploma and above	41 (21.6)
**Financial status**	
Good	20 (15.1)
Average	80 (60.3)
Weak	26 (19.6)
**Marital** s**tatus**	
Single	56 (19.9)
Married	134 (80.1)
**Use** **of** **hypnotics**	
Yes	137 (72.1)
No	53 (27.9)
**Job**	
Retired	163 (86)
Working	5 (2.4)
Employee	22 (11.6)

**Table 2.** The Relationships Between Pittsburgh Sleep Quality Items, Overall Sleep Quality, and Spiritual Health

Variables	1	2	3	4	5	6	7	8	9
**Subjective sleep quality**	1								
**Quality during the incubation period of sleep**	229, 0.006	1							
**Sleep duration**	0.198, 0.019	0.615 < 0.001	1						
**Quality of sleep**	0.237 < 0.001	0.966 < 0.001	0.494, 0.005	1					
**Sleep efficiency**	0.313 < 0.001	0.993 < 0.001	0.628 < 0.001	0.975 < 0.001	1				
**Use of sleeping medication**s	0.075, 0.381	0.294 < 0.001	0.26, 0.002	0.228, 0.001	0.229 < 0.001	1			
A**bnormal daily performance**	0.149, 0.079	0.254, 0.002	0.147, 0.043	0.233, 0.006	0.256, 0.002	0.3 < 0.001	1		
**Overall sleep quality**	0.273 < 0.001	0.304 < 0.001	0.383 < 0.001	0.431 < 0.001	0.333 < 0.001	0.261 < 0.001	0.206 < 0.001	1	
**Spirituality**	0.156, 0.32	0.061, 0.4	0.067, 0.35	0.155, 0.033	0.131, 0.07	0.066, 0.36	0.014, 0.85	0.149, 0.04	1

**Table 3.** Regression Analysis of Variables Associated with Sleep Quality

Independent Variables	Standardized Beta	P Value	R2
**Spiritual health**	0.209	0.04	
**Family**	0.053	0.52	
**Education**	0.015	0.95	0.417
**Job**	0.173	0.026	
**Income**	0.117	0.035	
**Marital **s**tatus**	0.112	0.045	
**Use of sleeping medication**s	0.106	0.044	

## 5. Discussion

We found that about 81.1% of patients had sleep disorders. In a study, Hanly showed that 52% of hemodialysis and 50% of peritoneal dialysis patients were highlights sleep disorders and indicated the changes in q as one of the disease challenges. He believed that sleep problems including difficulty falling asleep (67%), waking up at night (80%), waking up early (72%), and restless legs syndrome (83%) were the factors that affected the quality of life and jeopardized the overall health (21-23).

In a study, among clinical symptoms in hemodialysis patients seven were the most common, including insomnia (70-80% prevalence) and fatigue (70%), muscle cramps (57%), dyspnea (40%), pruritus (49%), headache (42%), and joint pain (44%), which were significant symptoms and required serious medical intervention. According to the patients, insomnia was mentioned as a troubling sign of the disease (13). Therefore, endangering physical health, spiritual health of patients poses a challenge. The results of study showed correlation between sleep quality and spiritual health of patients (13).

In hemodialysis patients, poor sleep quality can be seen with symptoms such as increased stress, anxiety, and depression (24, 25). In addition, sleep apnea syndrome, restless legs syndrome, and sleeping too much during the day as the main causes of insomnia are much more common in these patients than in the general population (24, 26). Poor sleep quality could lead to ongoing problems intensifying the physical complications which have additional negative effect on spiritual health of hemodialysis patients.

In this regard, Kimmel et al. found that 85% of hemodialysis patients had symptoms of sleep apnea with a higher rate than the general population (27). Therefore, sleep disturbances in hemodialysis patients should be considered by healthcare providers as one of the challenging problems. Due to direct impact of these disorders that could physically harm the patient and indirectly influence other aspects of health such as spiritual health, they have to encounter difficulties in their lives, which lead to increasing negative effects on quality of life. Another effective factor on patients' sleep qualities was too much sleep during the day. Perl and colleagues found that 77% of hemodialysis patients reported daily napping and 51% underwent involuntary sleep (23). In addition, researches showed that in patients with chronic renal failure, sleep quality is much lower than the general population (28). Moreover, studies showed that sleep disorders had a substantial negative impact on quality of life and affected the person's health status (25, 29, 30). Sleep problems as the important worriments were on the top of the signs and the problems experienced by this group as a major source of stressful factors with negative effects on quality of life. Furthermore, when stress symptoms are being checked, sleep problems are at higher tiers (31-33). Therefore, to improve the personal health and prepare these patients to enter the spiritual realm, enhancing their sleep quality is of great importance, because this problem affects other aspects of hemodialysis patients' lives and adds the frequency and severity of their problems.

Unruh found that difficulty sleeping or too much sleep during the day were two of the 12 main signs of these patients' problems (34). Perl et al. found that 42% of dialysis patients had sleep problems. Sleep disorders affected their daily activities and 21% stated that elimination of these symptoms will improve their life qualities (23). Therefore, it is necessary to design strategies for health promotion and care to this main concept, especially in home environments.

Considering the importance of mental health, meeting the human's basic needs is inevitable. Sleep is one of the most vital physical, mental and emotional needs of human and normal sleep is a sign of physical health and well-being. It reduces stress, anxiety and tension, enhances the body energy and mind focus, and balances the body to enjoy daily activities (35).

In hemodialysis patients, disease stresses increase the need for sleep. Comfort is one of the sleep necessities. During the disease, pain and discomfort emerge, which are caused by unusual physical statuses of medical interventions. However, sleep deprivation and related disorders are not the only causes of diseases and disorders. Sleep disruption can cause emergence of diseases manifestations. In longer sleep periods at the end of night, serious problems such as irregular heartbeat, angina, heart attack and stroke may occur. Neglecting these issues and lack of attention to sleep quality in hemodialysis patients can damage their spiritual health in addition to physical aspects, which might be followed with unpleasant problems (36).

Sleep disorders are frequently reported by patients with chronic renal failure and hemodialysis patients. The prevalence of sleep disorders in dialysis patients has been reported 50-80%. Furthermore, studies have shown that patients with chronic renal failure had lower sleep qualities compared with the general population. Therefore, early detection and intervention to improve sleep quality is necessary. Long-term sleep disturbances diminish people's health and normal living (37).

Sleep disorder symptoms must be considered by healthcare planners. Culleton et al. showed that 7.42% of hemodialysis patients had stress associated with moderate to severe sleep problems (38). Stress and emotional disturbances as subsequent side effects can damage patients' health statuses, which could affect their spiritual health and quality of life. Sleep disorders symptoms affect the patients' daily activities. In a study by Perl et al. on 165 patients with kidney diseases, difficulty in sleeping or too much sleep during the day were the two main concerns of 12 problematic factors (23). Therefore, to improve the quality of patients' daily functions, improved sleep quality is needed. Findings of this study showed a significant relationship between sleep quality of hemodialysis patients and their spiritual health. To be healthy during the disease or incidence of unfavorable conditions, emphasis should be given to all aspects of health. Physical health threat as a consequence of disease complications such as sleep disorders can endanger other aspects of health such as mental and spiritual health. Norum et al. in their study on cancer patients in Norway found that believing in God and praying could provide spiritual health. In these patients, having faith was positively correlated with quality of life and spiritual health (39).

High impact of the disease on all aspects of health is considerable. Healthcare intervention should be a holistic approach, considering all aspects of health including physical, spiritual, and mental health to support patients in facing the disease challenges. Finkelstein in a study on patients with renal failure proved that there was a direct relationship between spiritual health and quality of life. It was asserted that the prerequisite for improving the quality of life in patients with renal failure was paying attention to their spiritual health (11). O'Brien et al. in a study on 175 patients with breast cancer showed that religious women had higher spiritual health statuses. They mentioned that religion was the core motivation of their lives. In this study, approximately 81.1% of patients had sleep disorders (40).

As it was mentioned earlier, illness always is integrated into stress and psychological adversity. Considering the effects of sleep problems on mental disorders, disease management should be addressed for patients with special concerns. Hafen in a study entitled "The impact of attitudes and emotions on health" revealed that about 90% of patients, who coped with the stress of illness, benefited from religion and spirituality. They believed that religion brings them comfort. On the other hand, it was clear that stress of chronic illnesses can lead to loss of health, friends and family members. In societies that value people's fertility and young appearance, it causes more anxiety; therefore, they use religion to adapt to these kinds of stresses (10).

Regarding Iran's religious structure, patients, especially in later stages of their diseases, resort to holy people, praying and meditation to deal with illness stresses and pay special attention to their spiritual health. In this regard, healthcare providers must also understand the situation and be aware of spiritual health significance to help them along this way. Molassiotis et al. showed that patients with hematologic malignancies and higher education levels prayed more (41). In this study, education was considered as a predictor of sleep quality in hemodialysis patients. In a quality research conducted by Taleghani et al. female participants with cancer stated that resorting to a vow of pilgrimage and invoking the Imams had effects on mental relaxation and reduced fear of the disease. Patients believed that The Shia have Imams that can be resorted for relief and rescue. Women with breast cancer knew their religious beliefs as important factors for emotional support and believed that to feel more comfortable and to be able to move again they must rely on God's power (42). Generally, adequate rest, sleep and comfort of the patient as well as spiritual health care, are the main responsibilities of healthcare providers. Precise assessment of patients' previous sleep habits is very important for their mental and spiritual health (43). Studies have shown that religious beliefs and spiritual health during the disease trajectory are among the most important things, because in times of crisis or when other coping mechanisms are not efficient enough, people become more religious. In these cases, religious beliefs as strategy and lifestyle have provided good supportive source to individuals and they have been equipped with a variety of effective coping skills (35).

Moreira-Almeida et al. believed that patients who pay attention to their spiritual well-being were less depressed and anxious. Therefore, they were calmer and more comfortable in their lives, resulting in a better sleep quality (12). According to the results of the present study, paying attention to the quality of sleep in hemodialysis patients should be considered by healthcare providers. In addition, due to the close relationship of sleep quality with other health factors such as spiritual health, to improve the patients' sleep qualities, it is necessary to improve mental and spiritual health, leading to a better life quality.

### 5.1. Study Limitations

The limitation of this study was examining only one effective component on spiritual health of hemodialysis patients. However, this research was a prospective study paying attention to main concepts in hemodialysis patients which affect their life qualities. Investigation of other effective factors on spiritual health of these patients can be useful for future planning.

### 5.2. Recommendations for Further Studies

It is proposed to undertake interventions with qualitative approaches to explore the factors affecting the spiritual and overall health of hemodialysis patients. In this way, interventional programs could be designed to improve their life qualities. Sleep is one of the most vital physical, mental, and emotional needs of human with special importance in different aspects. Normal sleep is a sign of physical health, which can be influenced by pilgrimage, praying and resorting to Imams. The components of spiritual health, as factors in mental relaxation and reduction of the discomfort caused by the disease, were emphasized in this study. Hemodialysis patients' healthcare plans should be designed noticing these factors and components to promote their life qualities.
